# Supplementary figures and images for: Sclerostin small-molecule inhibitors promote osteogenesis by activating canonical Wnt and BMP pathways
Source: eLife. 2023 Aug 10;12:e63402. doi: 10.7554/eLife.63402 (PMC10431921; doi:10.7554/eLife.63402)

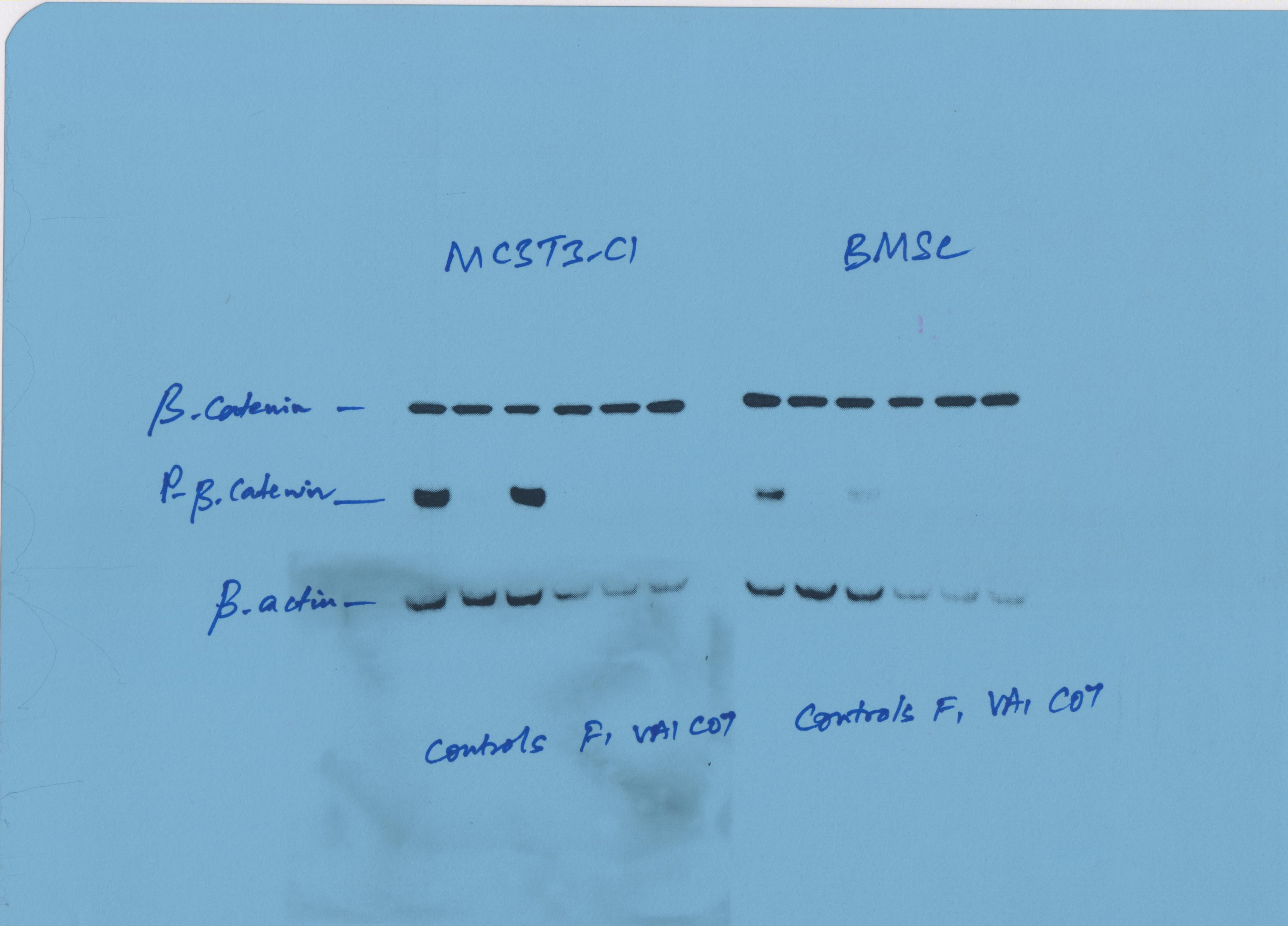

Supplement: Figure 2—source data 1. [file elife-63402-fig2-data1.zip › Figure 2-source data 1.jpg]

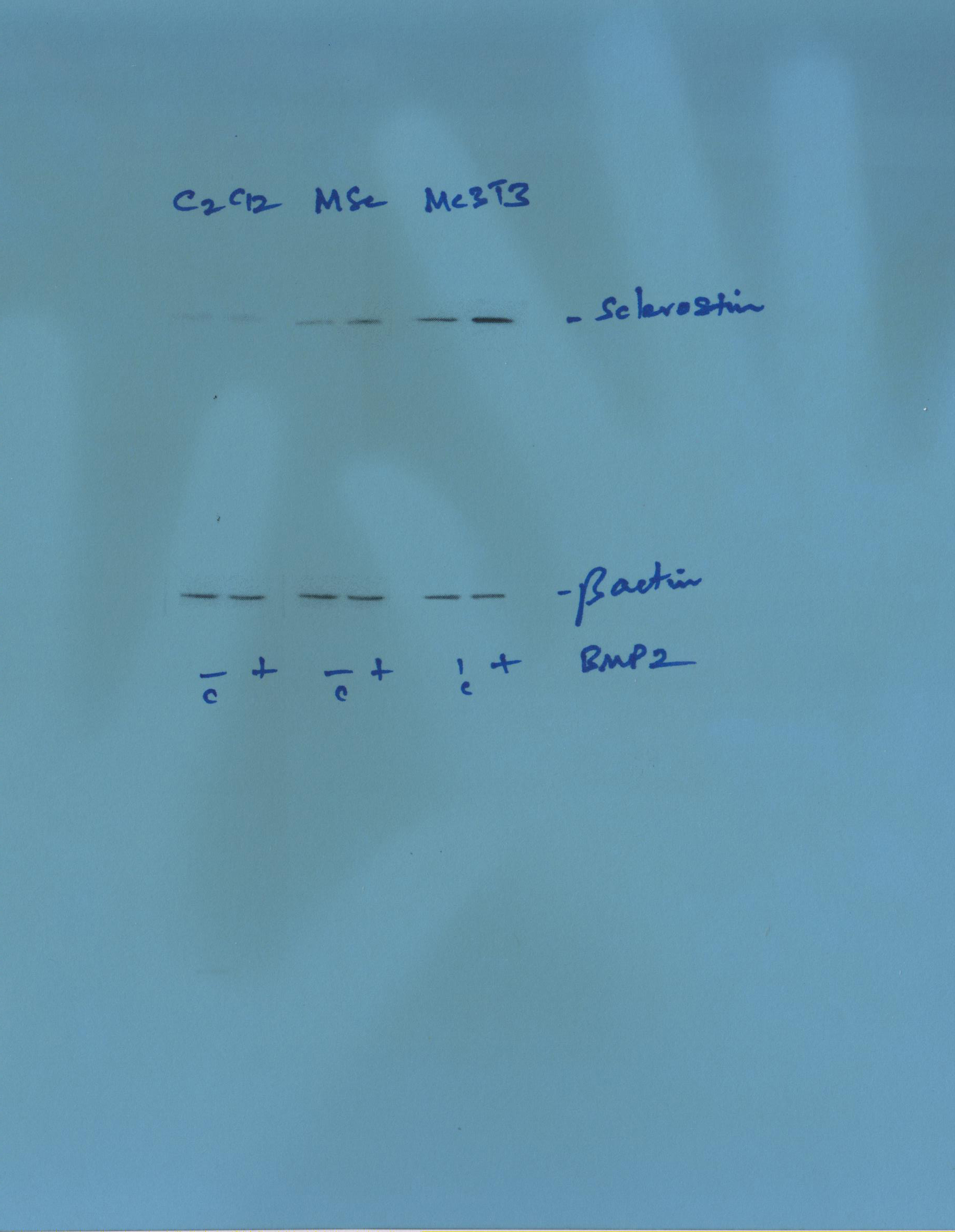

Supplement: Figure 2—source data 2. [file elife-63402-fig2-data2.zip › Figure 2-source data 2.jpg]

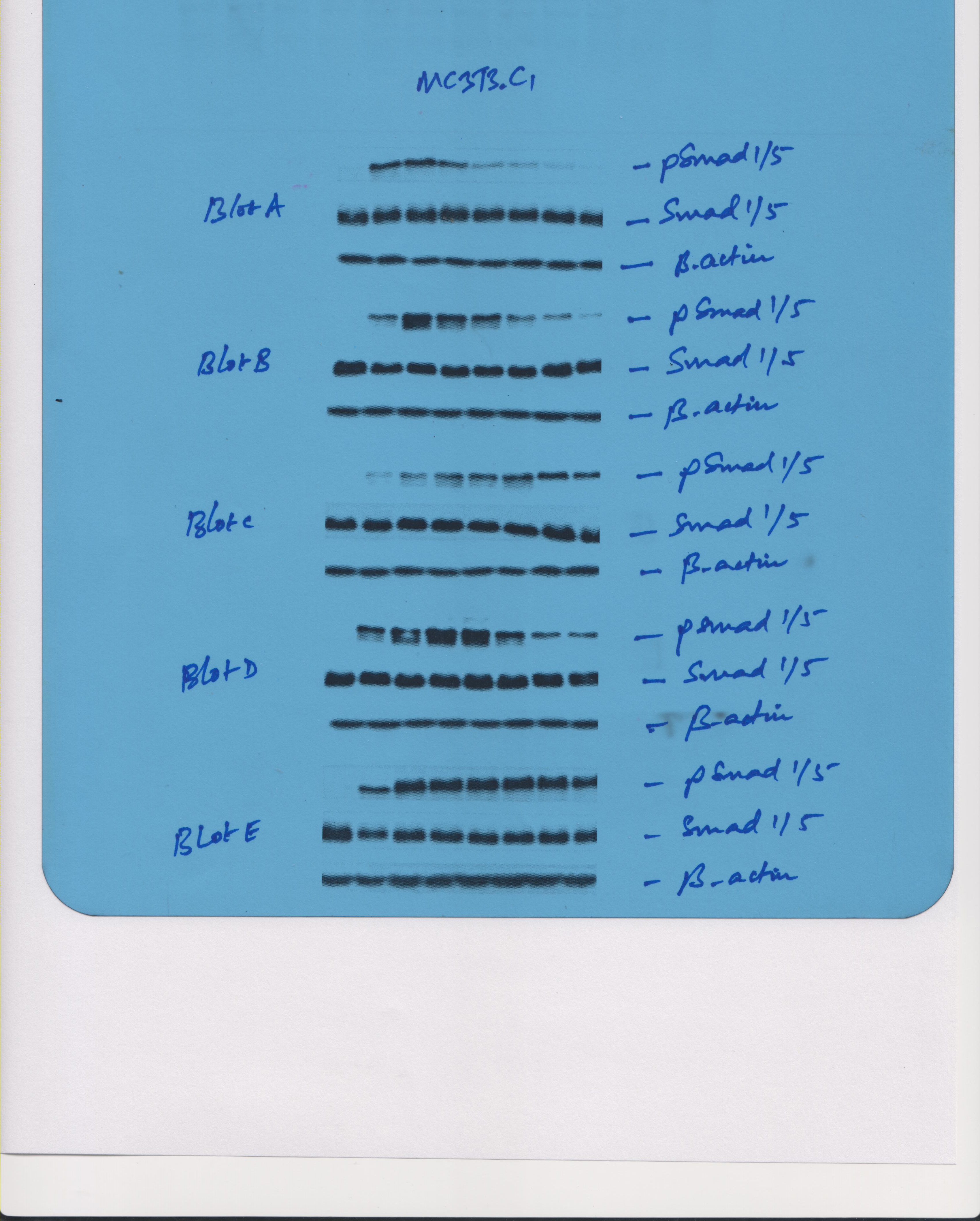

Supplement: Figure 3—source data 1. [file elife-63402-fig3-data1.zip › Figure 3-souce data 1.jpg]

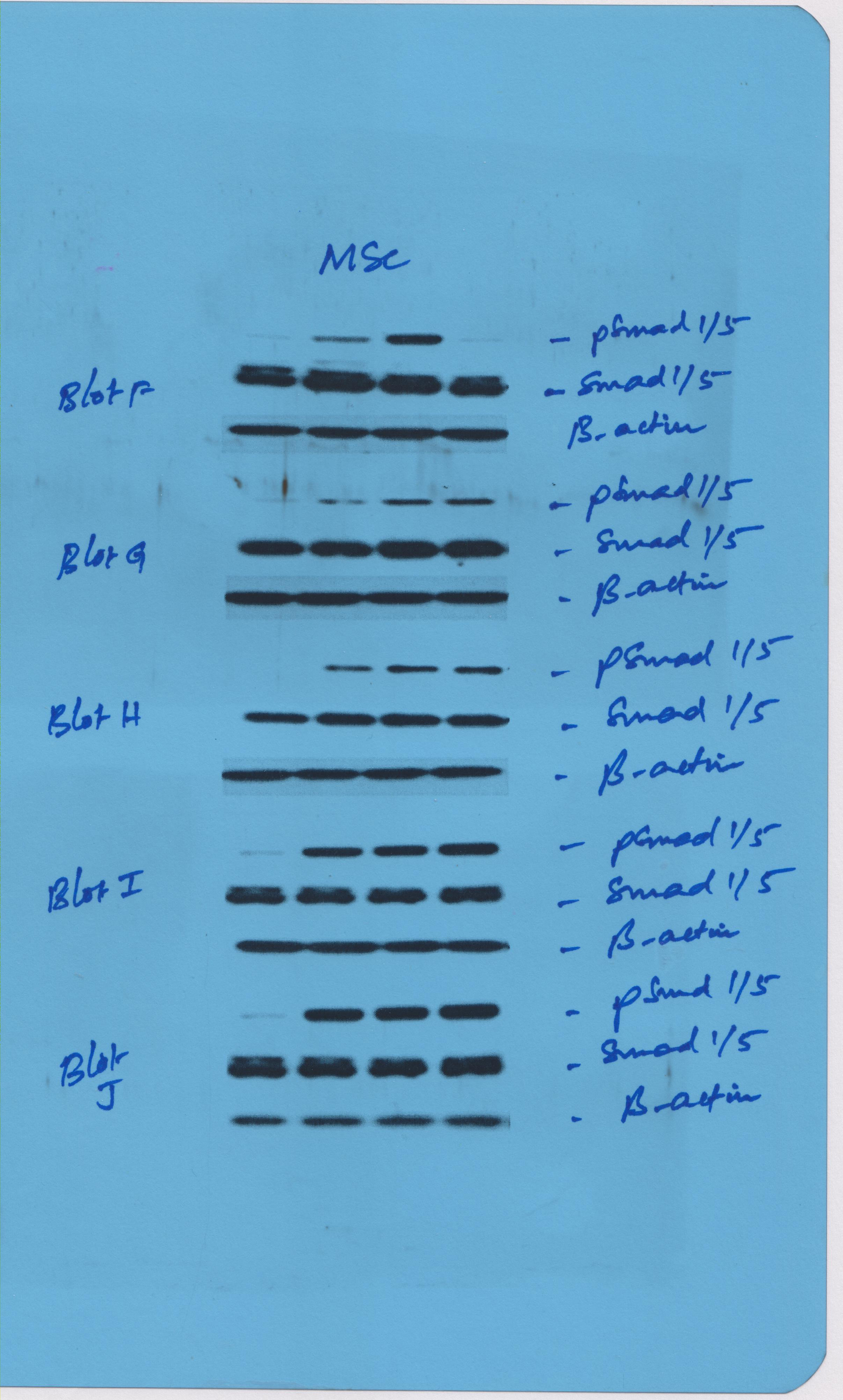

Supplement: Figure 3—source data 2. [file elife-63402-fig3-data2.zip › Figure 3-source data 2.jpg]

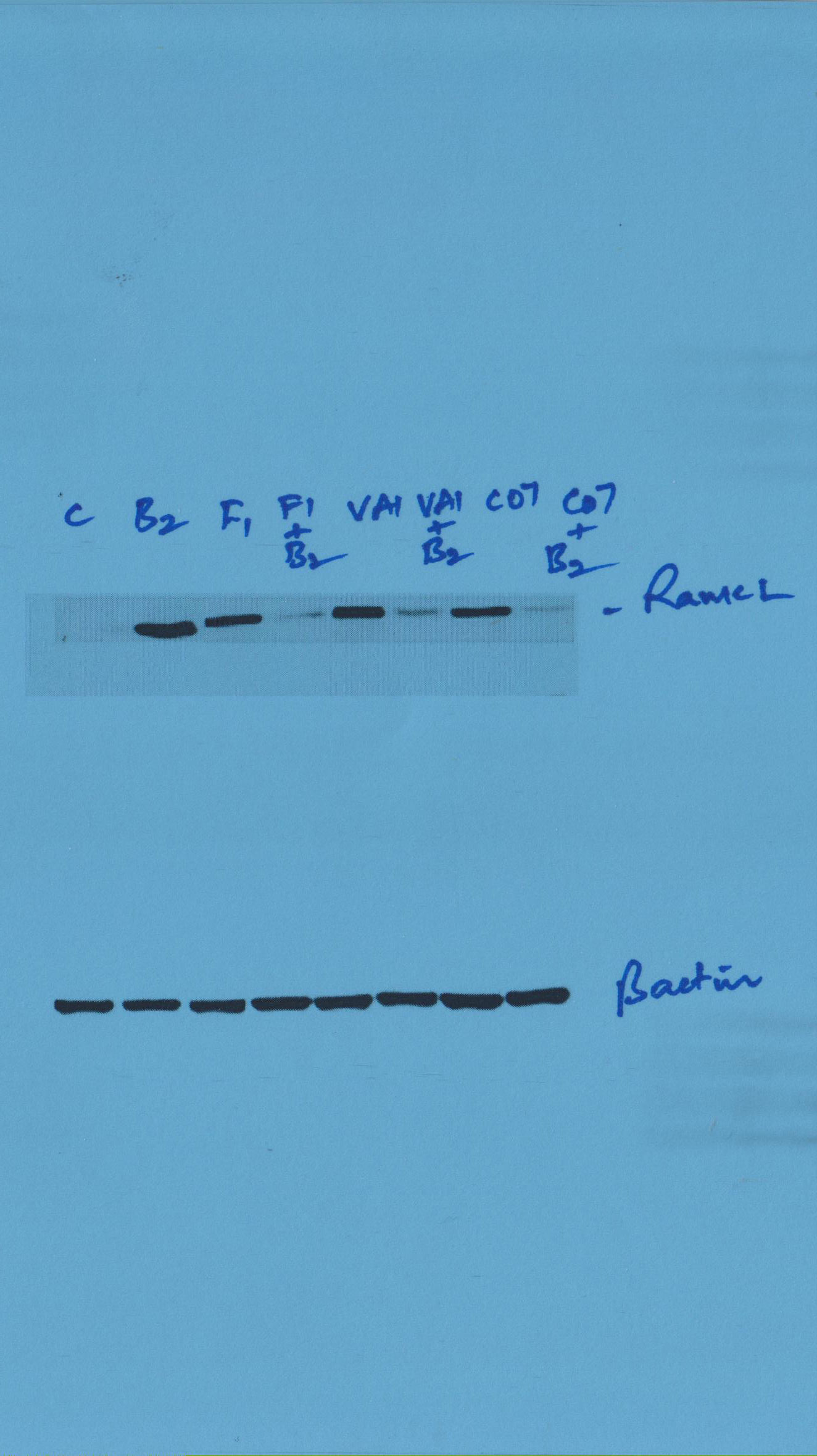

Supplement: Figure 6—source data 1. [file elife-63402-fig6-data1.zip › Figure 6-source data 1.jpg]

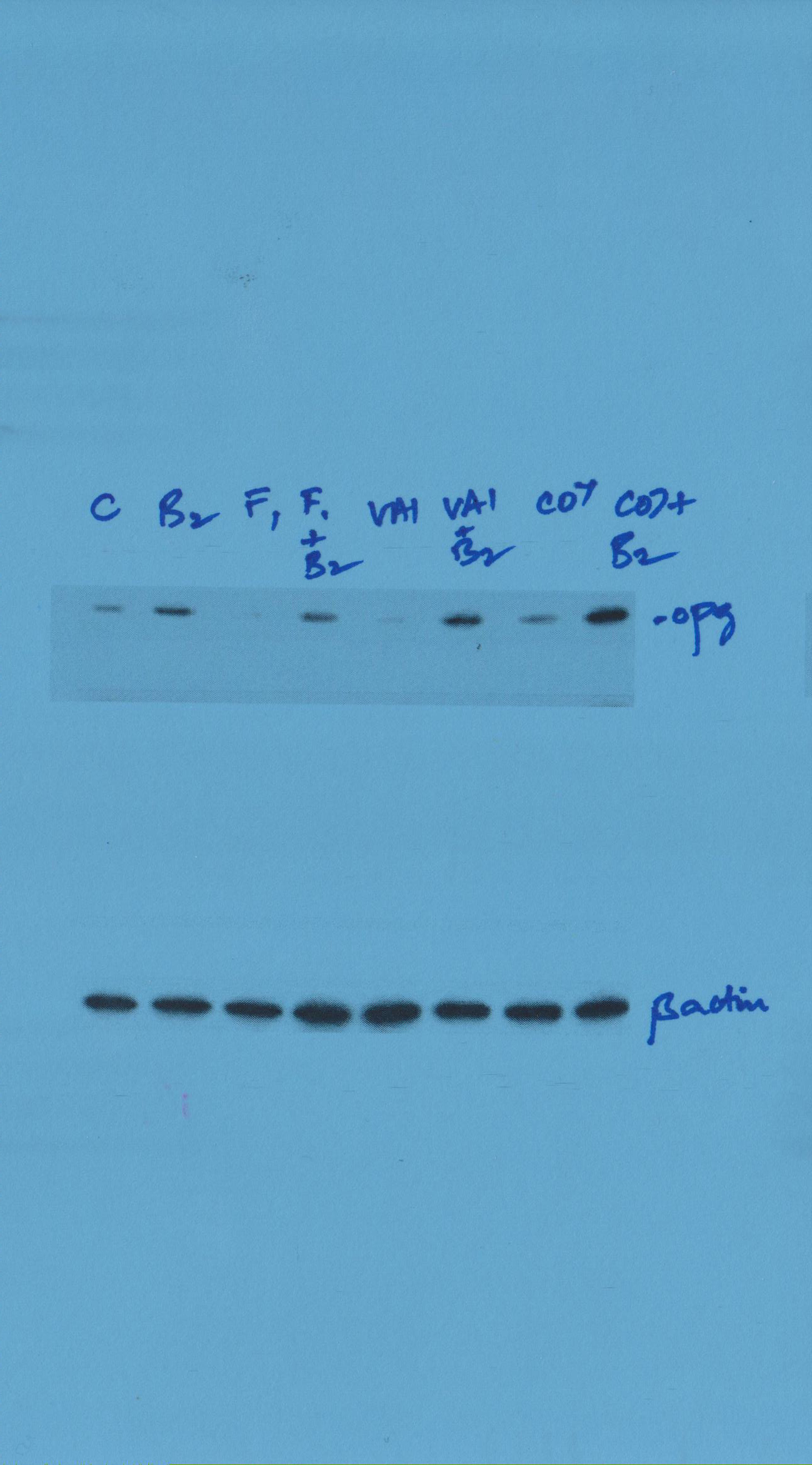

Supplement: Figure 6—source data 2. [file elife-63402-fig6-data2.zip › Figure 6-source data 2.jpg]
